# A hermeneutic analysis of delusion content from the casebooks of the Grahamstown Lunatic Asylum, 1890–1907

**DOI:** 10.4102/sajpsychiatry.v25i0.1263

**Published:** 2019-02-27

**Authors:** Rory du Plessis

**Affiliations:** 1 Centre for Ethics and Philosophy of Health Sciences, University of Pretoria, South Africa

## Abstract

**Background and objectives:**

This study sought to investigate the content of the delusions recorded in the casebooks of the Grahamstown Lunatic Asylum as a means to explore how the colonial context shaped or influenced psychopathology. To this end, the study aimed to (1) identify the sociopolitical events of the time period that were reflected in the delusion content presented by the patients and (2) pinpoint discernible patterns in the delusion content based on the race and gender of the patient. The study was delimited to the period of Dr T.D. Greenlees’ tenure as medical superintendent, 1890–1907.

**Methodology:**

The study sampled the casebook records of 400 patients. A qualitative analysis of the casebooks was followed by adopting a Gadamerian approach to hermeneutics. The analysis drew upon the clearly articulated method and step-by-step approach for Gadamerian hermeneutics outlined by Fleming, Gaidys and Robb.

**Results:**

The sociocultural and political events of South Africa during the turn of the 20th century had a marked influence on the content of the patients’ delusions. The South African War (1899–1902), the rinderpest epidemic of 1896–1898, diamond mining in Kimberley and the discovery of gold in the Witwatersrand were common features in the delusion content. Moreover, there is evidence of discernible patterns in the content of the delusions based on the race and gender of the patients.

**Conclusion:**

The study identified how the colonial context influenced the delusional content presented by the patients of the Grahamstown Lunatic Asylum. Of key significance is the fact that the study retrieved themes in the delusional content presented by black subjects that were silenced, omitted or censored from psychiatric texts published during colonialism.

## Introduction

In exploring the history of psychopathology in South Africa, Sally Swartz^1^ is interested in two key pivots:

On the one hand, this … [*is*] a history of disordered states of mind, and their recurring patterns over time, in South Africa. Thus, it is a history of the ways in which historical contexts enfold, shape and make insanity. On the other hand, it is a history of a science, of the men and women who defined, described and treated insanity. A history such as this also touches upon the madness of the state itself, the ‘something rotten’ of governments and their institutions. Hence, this is a history that must also touch on colonialism and the rifts and cracks of institutionalised racism. (p. 261)

In terms of the latter pivot, a wealth of scholarship has explored how the Cape Colony of the 19th century consisted of a network of psychiatric institutions that were underpinned by policies and practices of racial discrimination and segregation.^2^ One expression thereof was the building of separate institutions for black patients and white patients. This included Valkenberg Asylum (1891), which was established solely for white patients (by 1916 the asylum admitted black male patients and in 1919 black female patients),^3^ and Fort Beaufort Asylum (1894), which was reserved exclusively for black patients. Asylums that cared for white patients were allocated more resources. In contrast, the Fort Beaufort Asylum was primarily considered as an institution for cheap custodial care of black patients. For scholars like Sukeri, Betancourt and Emsley,^4^ historical studies of mental health care in the former Cape Colony are instrumental in revealing how the colonial and apartheid past laid a foundation for the ‘inequitable distribution of mental health services’ in the present-day Eastern Cape province of South Africa.

While the colonial history of psychiatric institutions has received much attention, Swartz’s former point, the investigation of how the colonial context shaped or influenced psychopathology, is still in its infancy.

Analysing the content of the delusions contained in the casebooks of psychiatric institutions offers a means to embark upon such an investigation as ‘delusions amplify social, political, economic and religious concerns and desires’.^5^

A growing body of contemporary scholarship in the history of psychiatry has not only established how the content of delusions is reflective of the sociopolitical context and the zeitgeist of the time but also how the content is influenced by a patient’s gender and race.^6,7,8,9,10,11,12,13,14^ For example, in David Wright’s^14^ analysis of the patients admitted to the Buckinghamshire Lunatic Asylum in Victorian England, he found that the content of women’s delusions:

tended to be associated with kinship, family and household, and personal health, a function of the cultural importance of these aspects to the changing social circumstances in which women’s social role became increasingly associated with the domestic sphere. Male delusions, by contrast were most often associated with work, status and property. Thus, the changing social and sexual role expectations of Victorian women and men mad were reflected and distorted in the content of their own false beliefs. (p. 153)

This article seeks to contribute to the aforementioned body of scholarship by analysing the content of the delusions recorded in the casebooks of the Grahamstown Lunatic Asylum (GLA), from 1890 to 1907. The analysis aims to (1) identify the sociopolitical events of the time period that were reflected in the content of the delusions presented by the patients and (2) pinpoint discernible patterns in the delusion content based on the race and gender of the patient.

The GLA (presently known as the Fort England Hospital) was established in 1875 in Makhanda, formerly known as Grahamstown. From 1875 to 1890, Dr Robert Hullah was the superintendent of the GLA.

Thereafter, Dr Thomas Duncan Greenlees (1858–1929) was appointed as the medical superintendent from 1890 to 1907. Greenlees wrote a considerable number of scientific articles, which were published in leading journals of the time, including the *South African Medical Record* (now known as the *South African Medical Journal*), the *Journal of Mental Science* (now known as *The British Journal of Psychiatry*) and the *American Journal of Insanity* (now known as the *American Journal of Psychiatry*). In addition, Greenlees published a number of articles in the newspapers that circulated in the town, and he was prolific in the talks and speeches that he delivered to the medical community and the general public of the town.^15^ This study is delimited to the period of Greenlees’s tenure for two primary reasons. Firstly, no casebooks were kept during Hullah’s tenure. Secondly, not only did Greenlees keep copious casebook records during his time of office, but the patient body of the GLA consisted of black and white subjects. By 1908, the GLA was reserved for white patients only. Thus, by locating the study to the period of Greenlees’s tenure, the content of delusions for black and white patients can be analysed for the presence of discernible patterns.

## Research methods

### Design and data collection

The study was a qualitative investigation of the delusions recorded in the casebooks of the GLA. The casebooks are archived at the Western Cape Archives and Record Service (WCARS). Permission to access the casebooks was obtained from the head of the WCARS.

### Setting

Over the period of investigation, the GLA admitted patients from across the reaches of the Cape Colony.

Thus, the content of the asylum’s casebooks features the broader cultural, social, political and economic backdrop of the Colony.

### Study population and sampling strategy

The GLA admitted, in terms of race and gender, a heterogeneous patient body that totalled, from 1890 to 1906, 2241 patients.^16^ In an effort to offer a representative casebook sample of the heterogeneous patient body of the GLA, purposive sampling was employed.^17^ A total of 400 cases were sampled for the study.

### Data analysis

The sampled casebooks were analysed by adopting a Gadamerian approach to hermeneutics.^18^ The analysis drew upon the clearly articulated method and step-by-step approach for Gadamerian hermeneutics outlined by Fleming, Gaidys and Robb.^19^ Very briefly, this entailed investigating every sentence of the casebooks to develop a meaningful understanding of the subject matter and the identification of themes and patterns in the delusional content. Once the themes and patterns were identified, the casebooks were repeatedly read to offer a rich and detailed interpretation of the delusional content. To this end, the analysis engaged in a hermeneutic circle in which the movement or interplay between the corpus of sampled casebooks to the records of individual patients allowed for the expansion of understanding as well as the contextualisation and comprehension of the themes and patterns.

## Ethical consideration

This article reports on the findings of a larger study^16^ that obtained ethics approval from the University of Pretoria’s Faculty of Health Sciences Research Ethics Committee. The article retains the anonymity of the Grahamstown Lunatic Asylum’s patients by using pseudonyms; however, in an effort to humanise the subjects, the patients are provided with full names rather than being identified by initials. In the article, a patient’s pseudonym is followed by an HGM citation that indicates the volume and page number of the relevant casebook accessed from the Western Cape Archives and Record Service.

The terminology used during the period under exploration permeates the article so as to convey the prevailing sentiments of the time, even though some terms are, or have become, demeaning and even derogatory in contemporary times.

## Results

### Sociopolitical events reflected in the delusional content

The sociopolitical events of South Africa during the turn of the 20th century had a marked influence on the content of the patients’ delusions. The South African War (1899–1902) (also referred to as the Second Boer War), the rinderpest epidemic of 1896–1898,^20^ diamond mining in Kimberley, the discovery of gold in the Witwatersrand, as well as the introduction and extension of electrification and telecommunication schemes across the Colony were common features in the delusional content of the patients.

To expand upon one of the events, the South African War weighed on the minds of the patients and punctuated the content of their delusions. There are numerous cases where the Colony’s civilian subjects were prey to delusions of guilt and persecution. Sean Fraser (HGM 7, p. 57), a farrier, considered himself ‘the cause of the Boer War’, as well as ‘the cause of all the trouble in S. Africa’. The homemaker Natalie Conroy (HGM 21, p. 37) was convinced that she was the cause of the war and all the ‘ills of the world’.

Kelebogile Sewela (HGM 5, p. 180), a 21-year-old from Beaufort West, was ‘certain that the Dutch wish to destroy the railways, and wants to prevent them’. For the interest that he professed in wanting to secure the railways, he believed that he was being watched by spies. A few casebooks illustrate the pain, suffering and anguish of individuals caught in the bloodstained battlefields and concentration camps of the war. Joseph Woodrow (HGM 7, p. 151) was a staff officer throughout the war. His medical certificates portray an individual who was exposed to the ‘hardships of active service’: he was unable to sleep at night, he appeared terribly thin and he was beset by a desire to take his own life. He laboured under a delusion that he was to be court-martialled, and that he was to be ‘shot or otherwise punished’. Kierin van Aarde (HGM 21, p. 88), an Afrikaner from Burgersdorp, was an inmate of a concentration camp. While incarcerated at the camp, two of her children died, which led to the onset of her mental affliction. At the end of the war, when she returned home, her distress did not abate, and her husband reported that she was violent towards him and would wander into the veld (open grasslands). On arrival at the asylum, the casebook indicates that she presented a delusion that while she was in the concentration camp, the wardens tried to poison her with ‘sheep dip’.

### Patterns in the delusional content

In the ensuing discussion, the study foregrounds how the delusions of grandeur presented by the GLA patients provide not only an index of how the colonial context influenced the content of delusions but also reveal the marked presence of a discernible pattern based on the race and gender of the patient.

Accordingly, the discussion explores how the race-gender profile of the patients yielded distinctive patterns in the delusional content.

#### Delusions presented by black subjects

For black patients, delusions of grandeur took two primary forms. In the first form, the delusions were based on or set in an African worldview.^21^ For black male patients, this was expressed in terms of professing vast tracts of land or plentiful stock of sheep, goats and cattle, and for some, wealth and importance was claimed by having ‘at least 1000 children’ (HGM 13, p. 109) or being a chief who has ‘24 wives and 50 children’ (HGM 4, p. 88). A recurrent theme in the delusions of grandeur presented by black women is a belief in either being pregnant or having given birth to an unconscionable number of children. Nomasomi Bila (HGM 17, p. 68) believed that she was a great personage and had 100 children, while Patricia Hanekom (HGM 20, p. 21) held that she was ‘better than other women’ and had 1000 children. Thandiwe Lebelo (HGM 18, p. 39) laboured under the delusion that she was advanced in pregnancy, and that Queen Victoria was to attend to her.

In the second form, there are multiple instances where the delusions of grandeur presented by black subjects featured the content and figures of the imperial and colonial context. For instance, Tinashe Dludlu (HGM 5, p. 172) was emphatic that he was ‘going to breakfast with the Queen and Oom Paul’ (the nickname of Paul Kruger who was president of the Zuid-Afrikaansche Republiek from 1883 to 1900), Sinalo Dandalo (HGM 18, p. 223) maintained that ‘she is Queen Wilhelmina’ (Wilhelmina Helena Pauline Maria was queen of the Netherlands from 1890 to 1948) and Lefika Gama (HGM 15, p. 14) proudly declared that his father was God, and that his mother was Queen Victoria.

A prominent theme in the content of the delusions of grandeur were black subjects believing themselves to be white. The black subjects’ claims to being white could be regarded as a desire for wealth, status and stature.^3^ The labourer Elias Manamela (HGM 15, p. 37) was convinced that he ‘is a white man’ who ‘has a large estate and lots of money’. The peasant Ican Makgabo (HGM 15, p. 82) informed the assistant medical officer, Dr Leslie, that he ‘is a white man’, and the general servant Marinda le Grange (HGM 16, p. 243), a woman of mixed race originally from the island of St Helena, asserted that ‘she is white having been painted black’.

A focal point in some of the cases where patients contended that they were white was not a desire for wealth but a disturbing contempt and scorn for black identity. The middle-aged housewife Anri Pretorius of mixed race (HGM 20, p. 32) declared that she was ‘going to beat all black people white’. Nqobile Zozo (HGM 15, p. 56), a general servant, called himself an Englishman and took on the name of his former employer, Mr Purdon. On admission to the GLA, he declared that ‘he is Mr Purdon and that he will never be a [*black*] again’.

As a tentative attempt to understand and interpret the anti-black sentiments of Anri and Nqobile, I turn to Frantz Fanon’s theory outlined in *Black Skin, White Masks.*^22^ Fanon identifies that a black subject’s ‘wish to be white’ is symptomatic of a racist society that ‘proclaims the superiority of one race’.^22^ In the contours of such a society, Fanon^22^ delineates that:

I begin to suffer from not being a white man to the degree that the white man imposes discrimination on me, makes me a colonized native, robs me of all worth, all individuality, tells me that I am a parasite on the world, that I must bring myself as quickly as possible into step with the white world […] Then I will quite simply try to make myself white: that is, I will compel the white man to acknowledge that I am human. (p. 73)

In this quote, Fanon contends that the racist suppression of black people into servitude and disseminating discourses of their inferiority to whiteness, leads black subjects to suffer feelings of ‘insignificance’.^22^ The ‘only way out’ of such feelings is for the black subject to emulate white people.^22^ Such a quest steers black subjects to become obsessed with ‘attracting the attention of the white man’, and to concerns ‘with being powerful like the white man’.^22^ Thus, a black subject is confronted with a dilemma, namely, to *turn white or disappear* (italics in original).^22^ It is this dilemma that Fanon, over the course of his book, seeks to dismantle.

By adopting a Fanonian reading, which seeks to examine the trauma and dehumanisation engendered by colonialism and racism,^23^ the sentiments professed by Anri and Nqobile can be understood in terms of how their lives were subjugated by colonial regimes and how their personhood was abased by colonial discourses that propagated their perceived inferiority, weakness and deficiency. Such acts of dehumanisation, racism and psychic suffering inflicted by colonial domination possibly resulted in their renunciation of a black identity, and efforts to imagine or make themselves and others white people.

In contrast to the delusions where black subjects imagined themselves as white, the casebooks also contain several cases where a black subject’s delusions express a passionate condemnation of white supremacy, as well as a simmering anger at colonial policies. Shima Thamae (HGM 5, p. 191) declared that he was sent by God to preach to black people to repent of their sins and to ‘keep out of the white man’s clutches’. In an indictment against the Colony’s forced dispossession of black subjects from their land, Vuyo Sondiyazi (HGM 5, p. 122) fervently voiced that ‘he is the Chief Sandile sent down from Heaven to claim land taken from him by Government’.

Illuminating in Vuyo’s statement is his reference to Chief Mgolombane Sandile Ngqika (1820–1878). Chief Sandile led the Xhosa armies in several of the Cape Frontier Wars (fought between 1779 and 1879).^24^ He was respected by his people, gained a reputation as a hero and was revered by the other Xhosa chiefs.^25^ In this way, the content of Vuyo’s delusion reveals that he was conversant in the biographies of black leaders who had stood up to the injustices of colonialism, and who refused to submit to claims regarding the superiority of white rule. Thus, it may be plausible to suggest that Vuyo’s invoking of Chief Sandile spoke against the oppression and subjugation of black subjects that the colonial project engaged in.

#### Delusions presented by white subjects

For white men, delusions of grandeur took the form of possessing great wealth, being successful in business ventures and believing in their elevated importance, by being bestowed with ranks and titles, as well being elected into exclusive fraternities and societies. While most white men professed to being royalty, in particular the King of England, there was also a significant number of men that professed to being some of the influential figures that dominated the political and financial spheres of the 19th century Cape Colony. One such figure that takes centre stage in the delusional content of several patients is Cecil John Rhodes.

To understand and interpret this phenomenon, I turn to the work of Laure Murat.^26^ In the Parisian asylums of the 19th century, Murat identifies that the figure of Napoleon came to dominate the content of the delusions presented by the patients. This observation leads Murat to ask, ‘why and in what way did the image of Napoleon lend itself more willingly to megalomania than the figure of, say, Charlemagne or Louis XIV?’^26^ After embarking on an insightful and thorough investigation, Murat offers the following conclusion:

If any role is going to be adopted, it may as well be that of the most powerful, most feared figure – who also happened to be the most recent one. Napoleon was the image par excellence of a superman, the very symbol of modern omnipotence and domination. Fair enough. Yet the singularity of this case also rests on another feature that sets Napoleon apart from every monarch who preceded or followed him. Compared with kings who incarnated the dynastic past of an age-old monarchy based on divine right, Napoleon was the Usurper, the little corporal from Corsica who came to rule Europe all on his own. His legitimacy was not *inherited* but *won* through political genius and armed might. Whereas a king was crowned by the grace of God and the luck of birth, Napoleon assumed his crown on his own authority […]. It matters little whether he was a savior or a dictator, adored or hated: in the eyes of his contemporaries, Napoleon represented the unique case of an adventurer who climbed to the head of a country all on his own. In the end, he was a perfect example of the American ideal of a self-made man. (p. 132, [*italics in original*])^26^

Drawing on Murat’s hypothesis, I suggest that Rhodes’ success encapsulated the myths and tropes of colonialism for white immigrants and settlers. Rhodes (1853–1902) was the son of a vicar and grew up in Hertfordshire, England. As he was a sickly child, his family sent him to South Africa in the hope that his health would improve. He arrived in South Africa at the age of 17. A year later, he entered the diamond trade of Kimberley. In the decades to follow, he became an ‘African Colossus’,^27^ who rapaciously seized 2.6 million square kilometers of Africa and brought it under imperial rule, and after gaining near control of the world’s diamond market, was described as ‘the first of the new Dynasty of Money Kings [who are] the real rulers of the Modern World’.^27^ Rhodes, without a royal lineage, entered the Colony and became a self-made man, whose reach, influence and image extended around the globe.

For recent immigrants to the Colony and settlers alike, Rhodes must have encapsulated the promises and potential of life in the Colony. For example, Conor Walsh (HGM 8, p. 91), a recent Irish immigrant to the Colony and engaged as a diamond miner in Kimberley, was remanded in custody following a violent and abusive outburst. During his detention, he was certified to be insane and arrived at the GLA claiming to be Cecil Rhodes. For some of the male patients at the asylum, like Conor, their humble beginnings in the British Isles and their immigration to the Colony was similar to Rhodes’s background, and thus they may have revered and mythologised the image of Rhodes and aspired to his success.

Professing to be a queen and of noble lineage was an intriguingly dominant theme in the delusions of grandeur presented by white women. Kate Willis’ (HGM 16, p. 117) medical certificates indicate that she claimed that she was the Queen of England. On admission to the asylum, Kate declared that she was the Queen of Cape Town. The domestic servant Hannah Baron (HGM 21, p. 111) believed that she had ‘blue blood’, and she would often ‘treat others with contempt’. A peculiar feature that can be observed in a portion of the delusions of grandeur was the women insisting that they were pregnant.^12^

In the delusions of grandeur presented by women, there is an absence of the content that predominated in white male delusions: success, status and importance achieved by business ventures, membership of elite fraternities and claims of being influential figures in the Colony’s political affairs. In this sense, the delusional content presented by white women remains fixed to the valuations of female success in the Victorian era. To elucidate, the pregnancy content within the delusions of grandeur is a mirror of the dominant views of patriarchal culture that valued women in the roles of maternity and motherhood and restricted them to the confines of the domestic realm. A chance to envisage life beyond such a realm was possibly encapsulated in the lives of noble birth and being crowned a queen. Thus, the content of the women’s delusions echoed Victorian gender roles, rather than expressing an alternative worldview, one where dominant patriarchal norms were dislodged and women claimed success by conceiving inventions, amassing great wealth on the stock market and, without the benefit of noble birth, becoming a self-made woman.

## Discussion

Roy Porter^28^ astutely observes that ‘there is no more splendid cache of psychopathological material than the delusions recorded over the centuries by the insane’. Yet, it is important to acknowledge that the lines of enquiry offered to the researcher are shaped by the medium in which the delusions were recorded. Delusions recorded by patients in their letters and diaries offer the researcher with insight into the voice of the patient and their personal account of psychosis.^7,29,30^ In contrast, the delusions recorded in the casebooks of psychiatric institutions are often only curt entries logged by the doctor. Although casebooks offer the researcher only an epigrammatic vignette of the content of delusions, once the individual vignettes are strung together, shared patterns based on the race-gender profile of the patients become discernible. Thus, in the exploration of the GLA’s casebooks, by seeking to string together the vignettes from the same demographic profile of patients, the study provides a portrait of the shared tropes, patterns and motifs in the delusional content.

In the delusions of grandeur presented by white men, a significant number of men professed to be some of the influential political figures that dominated in the Cape Colony, including Cecil Rhodes. For white women, there is a surprising paucity of references to imaginings of being successful in business and claims of being one of the prominent political leaders of the Colony. Instead, their delusions were characterised by achievements confined to the domestic sphere, triumphs of pregnancy and birth, and royal status. Catharine Coleborne^10^ insightfully asserts that the content of delusions ‘can provide more information about gender and behaviour, along with ideas about colonial life’. Following this line of reasoning, the delusions presented by the women of the GLA may offer some perspective on the extent to which the patriarchal society of the Colony had scoured their consciousness of ambitions, visions, thoughts and ideas that departed from maternal and marital modes of existence.

The delusions of grandeur presented by black subjects took two primary forms. In the first form, the content of the delusions was based on the markers that African societies of the 19th century held to be indicative of wealth, status and achievement. The delusions referred to abundant animal stocks, being a chief and having multiple wives and children. In the second form, the delusions abounded with content from the colonial context. While some black subjects imagined forms of grandeur, by either dining with royalty or being the progeny of Queen Victoria, others claimed eminence and wealth by believing they were white individuals with large sums of money. In some instances, a black subject’s desire to be white was linked to an abhorrence of black identity. For these black subjects, a Fanonian reading of their desire to be white while simultaneously rejecting black identity foregrounds the way colonial rules, practices and discourses had enchained their lives to feelings of insignificance. Yet, there were also delusions that were a protest against colonial rule. In particular, Vuyo Sondiyazi’s (HGM 5, p. 122) passionate assertion that ‘he is the Chief Sandile sent down from Heaven to claim land taken from him by Government’ can be read as a bold act of voicing detestation and scorn for the colonial policies that dispossessed black subjects of their land. Furthermore, it is plausible that Chief Sandile was invoked in Vuyo’s delusions as he was an icon who opposed and defied imperial domination, as well as an icon who could liberate black subjects from feelings of inferiority and insignificance.

The casebooks of the GLA provide a detailed inventory of the delusions of grandeur presented by black subjects that featured the content and figures of the imperial and colonial context; however, the psychiatrists of the Colony did not publish such findings. In several of Greenlees’ publications,^31,32,33^ he provides examples of the cases of delusions presented at the GLA but none of these cases included black subjects. While Greenlees’ texts were mute on the content of delusions presented by black subjects, they were vociferous in claiming that black subjects had an ‘infantile mind’^34^ and the type, form and content of their psychopathology was ‘simpler’^34^ than in white subjects.^35,36^ Pursuing this line of thought, John Conry,^37^ the medical superintendent of the Fort Beaufort Asylum, professed that:

… the Native seldom expresses delusions of an exalted character, more frequently his delusions concern stock, *i.e*., cattle, sheep, etc., that he possesses, or that have been taken from him, or that he has a number of wives and children when such is not a fact. His delusions follow the line of his normal mentalisation. (p. 34)

Conry thus omits the delusions of grandeur presented by black subjects that contain colonial symbolism to peddle a racist conception of the African mind as inferior, weak and primitive.^34,35,36^ This study has therefore retrieved the delusional content that was silenced, omitted or censored from psychiatric texts published during colonialism. In doing so, the study offers not only a countervail to the central discourses of colonial psychiatry but also identifies how the colonial context influenced the delusional content presented by the GLA’s black patients.

## Conclusion

The study identified how the colonial context influenced the delusional content presented by the patients of the GLA. Moreover, the study retrieved themes in the delusional content of black subjects that were silenced, omitted or censored from psychiatric texts published during colonialism. The omissions and inconsistencies between the casebooks and the published records of an asylum are for Jonathan Andrews^38^ an important tool for a researcher to identify the ‘areas of bias and censorship’ that were held by a psychiatrist. In this way, the article foregrounds how Greenlees and Conry’s published texts engaged in acts of censorship and erasure in favour of affirming colonial paradigms and discourses.
